# Effects of Light Pollution on the Early Life Stages of the Most Abundant Northern Red Sea Coral

**DOI:** 10.3390/microorganisms8020193

**Published:** 2020-01-31

**Authors:** Raz Tamir, Gal Eyal, Itay Cohen, Yossi Loya

**Affiliations:** 1 School of Zoology, George S. Wise Faculty of Life Sciences, Tel Aviv University, Tel Aviv 6997801, Israel; yosiloya@gmail.com; 2 The Interuniversity Institute for Marine Sciences in Eilat, Eilat 8810302, Israel; 2itaycohen@gmail.com; 3 ARC Centre of Excellence for Coral Reef Studies, School of Biological Sciences, University of Queensland, St. Lucia, QLD 4072, Australia; gal4596@gmail.com; 4 The Mina and Everard Goodman Faculty of Life Sciences, Bar-Ilan University, Ramat Gan 5290002, Israel; 5 Department of Oceanography, The Institute of Earth Sciences, The Hebrew University of Jerusalem, Jerusalem 9190401, Israel

**Keywords:** anthropogenic disturbance, coral recruitment, coral reefs, ecosystem management, fluorescent lights, LED lights, light pollution, photosynthesis

## Abstract

The growth in human population along coastal areas is exposing marine environments to increasing anthropogenic light sources. Despite the potential effects of this modern phenomenon, very few studies have examined its implications for corals. Here, we present a long-term study of coral early life stages under light pollution conditions at night. Coral larvae were collected from *Stylophora pistillata* colonies, and then settled and grown under experimental conditions of two different common city lighting methods (fluorescent or LED). Effects of the artificial lighting on the coral settlement success, survivorship, growth rate, photosynthetic efficiency, and calcification rate were examined over a period of one year. The control exhibited ~30% higher settlement success compared to the two light treatments, while under the light treatments corals showed higher survivorship, growth, and calcification rates. In addition, an indication of damage to the photosynthetic system was found in the light-polluted corals, which was reflected in their photosynthesis efficiency parameters: i.e., lower maximum light utilization coefficient (α), lower maximum potential photosynthetic rate (P_max_), and lower photosynthetic maximal quantum yield (F_v_/F_m_). Our findings provide evidence of the potential adverse effects of artificial lighting methods on the natural environment of coral reefs. We conclude that the use of the LED lighting method has high interference potential for the early life stages of corals.

## 1. Introduction 

Coral reefs, which are highly sensitive and complex ecosystems, are continuously exposed to a variety of both direct anthropogenic disturbances: e.g., sewage, nutrient enrichment, and diving activities [1]; and indirect ones: e.g., water thermal stress and acidification [2,3]. In many cases, such disturbances have been found to be a key factor in contributing to changes in coral-reef community structure [1]. From an ecological perspective, the light-quality regime (intensity and spectrum composition) is a crucial factor in affecting coral settlement [4,5] and survivorship [6], and hence strongly determines recruitment success [7]. Different responses of coral planulae to light quality and quantity have led to a species-specific spatial settlement in the reef [8]. Consequently, due to the corals’ light reaction mechanism, even the slightest change in light intensity and composition, as a result of artificial changes (i.e., light pollution), may alter the settlement pattern of different species, and directly affect the local and spatial community structure [4]. 

Previous studies have indicated that even low levels of light pollution may have an impact on the daily changes in moonlight that occur during the lunar cycle, and which are essential for maintaining the normal lunar periodicity; and, as a result, affect the coral’s biological clock (e.g., gene transcription) [9,10]. Using LED lighting as the light source, Boch et al. [11] demonstrated that the major driver of spawning on a given night of the lunar cycle appears to be that of a critical threshold, determined by lunar photoperiod cues and possibly also wavelength-dependent. Those mechanisms were found to be synchronized in accordance with the detection of moonlight by blue-light-sensing photoreceptor cryptochromes, which absorb mainly blue light [12]. Tamir et al. [13] demonstrated that in light-polluted areas, artificial light intensity at night can be higher than that of full moonlight. Thus, the combined accumulation of natural and anthropogenic stressors can result in adverse effects on coral reproduction [9,14], and may lead to a diminished or unsynchronized supply of coral planulae [15]. This may in turn cause a negative cascading effect on larval dispersal and recruitment in coral reefs [14]. 

Symbiotic dinoflagellates of the family Symbiodiniaceae are known to have a fundamental mutualism with many reef invertebrates, notably, stony corals [16]. This relationship was found to be influenced by differential adaptation to light conditions [17]. Frade et al. [18] found a strong functional within-colony uniformity in symbiont diversity due to variability in physical factors (e.g., irradiance, light spectral distribution, temperature) even among closely-related coral species. Byler et al. [19] presented evidence that juvenile colonies of *Stylophora pistillata* may utilize both vertical and horizontal symbiont acquisition strategies. Hence, changes in light conditions due to artificial sources may alter this natural pattern and affect the composition of the symbionts acquired at a coral’s early life stages (i.e., as larvae or juvenile colonies). However, this hypothesis has not yet been validated. Recently, Rosenberg et al. [20] demonstrated variability in chlorophyll (Chl-*a* and Chl-*c2*) concentration in LED-illuminated *Acropora eurystoma* corals, which exhibited higher values than the control ambient samples.

However, little is currently known about coral recruitment dynamics (e.g., settlement and post-settlement survivorship and growth) under a continuous change in the natural light regime at night, due to artificial lighting. Moreover, compared to daylight [8], the potential for habitat selection by larvae in the presence of artificial light at night is largely unknown.

Following the increase in the human population along coastal areas in recent decades, the natural nocturnal physical conditions have been altered by means of artificial lighting [21]. This spread of electric lighting has been shown to be a major perturbation to the natural nocturnal light regime [22]. The potential impact of such nocturnal light is indicated in the term “ecological light pollution” [23]. For a variety of reasons, this phenomenon is rapidly increasing in coastal areas [21].

The potential of artificial light at night-time to disrupt coastal and marine environments [22] has only recently become widely recognized as an environmental issue [24]. Moreover, insufficient attention has been given to date to its potential impact on coral reefs in general and on coral initial life stages in particular. 

The different lighting methods that characterize a city’s illumination result in variability in the spectrum and intensity of the artificial light sources [25]. Tamir et al. [13] showed that as a result of water clarity and the proximity of artificial light sources to the coastline, light pollution could be detected down to 30 m depth in the northern Red Sea. The significant change in the night-time light regime in the shallow depth zone is therefore expected to have a crucial effect on the northern Gulf of Eilat/Aqaba (GoE/A) coral-reef ecosystem. Nevertheless, despite the potential significant impact of light on coral settlement, survival, and distribution, insufficient attention has been paid to date both to the role of light pollution in dictating the settlement and zonation assemblages of corals, and to light pollution effects on such crucial mechanisms to corals as photosynthetic efficiency and calcification rates.

Considering the limited studies engaging with these issues to date, our current data on this phenomenon’s potential impact provide a novel and better understanding of these issues. Here, for the first time, we present a long-term experiment examining the effects of two artificial light sources on a coral’s initial life stages, as well as the potential effects of artificial light on a coral’s basic physiological systems and processes (e.g., photosynthesis and calcification).

## 2. Materials and Methods

### 2.1. Ethical Statement 

This study was conducted in accordance with the Israeli Nature and Parks Authority approval to work with animals, and in accordance with the conditions of permits No. 2017/41560 (1.2.2017-30.6.2017) and No. 2017/41683 (10.5.2017-10.5.2018).

### 2.2. Planulae Collection

Planulae of the coral *Stylophora pistillata* were collected in front of the Interuniversity Institute for Marine Sciences in Eilat, from March to June 2017. This abundant Indo-Pacific and Red Sea coral species was chosen because it is one of the most studied and common (in shallow water - < 30 m) coral species in the GoE/A [26,27]. Planula traps were used to collect planulae from 10 large colonies (20–30 cm in diameter) in shallow water (2–5 m depth). Each trap comprised a floating plastic vessel connected to a 120-μm plankton net via a plastic funnel at the top. The traps were deployed at sunset and collected early the following morning, placed in plastic containers with seawater, and brought immediately to the laboratory for counting. Each net was first washed with seawater and then transferred from the traps’ plastic containers into Petri dishes. A pooled random mix of planulae from the ten colonies was counted using a glass Pasteur pipette. In order to determine the exact proportion of planulae settled, an exact known number of larvae were introduced into individual 2-L glass vessels (50 planulae per vessel). Each vessel contained a 10 × 10 × 1 cm ceramic terracotta tile previously conditioned for six months in an open seawater run-through system under the natural ambient conditions of Eilat. 

### 2.3. Experimental Setup 

The experimental setup contained open seawater run-through system tables. The two treatments and the control were divided into three separate tables with no water exchange among them. A constant flow of the same seawater was supplied to all treatments in parallel. Water temperature was continuously monitored by thermometers (HOBO, Onset Computer Inc., Bourne, MA, USA) to assess variations in temperature of the seawater flowing through the open system. In order to simulate the existent artificial light, two light treatments that represented the most common city lighting methods were used (see Tamir et al., 2017 [13]): ‘Yellow’ light—fluorescent lamp (EL-PAR38/WW, 25 W, 2700 K, Eurolux, Cape Town, South Africa); and ‘White’ light—LED lamp (PAR38—8 W, 2900 K 230 V, 635 Lm, Eurolux, Cape Town, South Africa). A control treatment of ambient conditions during the night (moonlight only), represented the unpolluted area (1 × 10^−6^ μmol photons m^−2^ s^−1^, after Tamir et al. 2017 [13]). All treatments were equally exposed to the ambient daylight at 10 m depth (intensity peaks at 600 µmol photons m^−2^ s^−1^). In order to simulate the artificial lighting conditions at night, the lamps were connected continuously to a photocell sensor (220V/AC-240V/AC, 50Hz). In order to simulate the light intensity at the polluted reefs [13], two neutral density filters (0.6ND—LeeFilters, Hampshire, UK), were placed under each lamp (LED and fluorescent) to reduce the projected light to 0.8 µmol photons m^−2^ s^−1^. 

### 2.4. Settlement, Survivorship, and Growth Rate 

The experiment was divided into two stages—settlement and survivorship. During the first stage, four vessels were deployed for each of the three treatments (LED, fluorescent, Yellow, and control), without any water exchange between the vessels and tables. This was in order to prevent any external planulae from settling on the experiment tiles. We repeated the same process three times (4 x 3—i.e., 12 replications for each treatment). The water in the vessels was replaced every 48 h via a plastic funnel with a 120 μm plankton net, and the planulae that had settled on each tile were counted. After six days, when no swimming planulae could be observed in the water, the settlement tiles were moved to the open-system water tables for the second stage: growth rate and survival. During this stage, the tiles remained throughout the year in the water tables corresponding to each light treatment. Excluding the artificial lights at night, the run-through tables were constantly supplied by similar conditions (i.e., daylight, water temperature, and flow rate), for all treatments in parallel (Supplementary Figure S1).

After one year under the experimental conditions, the recruited corals were counted and measured in order to determine growth rate and survival. The survivorship percentage was calculated from the number of colonies recorded on each tile after one year divided by the number of juvenile corals that had settled on each tile at the end of the settlement period. To determine growth rate, we measured the projected surface area (cm^2^ year^−1^) for each colony using Photoshop software (Photoshop CS6, Adobe Inc., San Jose, CA, USA).

### 2.5. Photosynthesis vs. Light Energy (PE) and PSII Efficiency 

#### 2.5.1. PE Curves 

Each treatment tile was incubated in a sealed 800 mL acrylic metabolic chamber. The chambers were placed in a temperature-controlled bath, with constant water flow at 23 °C (i.e., mean ambient seawater temperature), maintaining a constant temperature and with a magnetic stirrer maintaining water movement inside the chamber. The tiles were subjected to increasing light intensities (0, 10, 30, 100, 200, 400, 550 µmol photons m^−2^ s^−1^) using a full-spectrum metal halide lamp (400 W, 5000 K, 50 Hz, Golden-Light, Netanya, Israel), for 20 min at each intensity. Light intensity (E) was recorded using a LI-COR LI-250A light meter (Li-Cor, Inc. Lincoln, NE, USA). Oxygen evolution was monitored continuously using Fire Sting O_2_ Optimal Oxygen Meter (Pyroscience, Bremen, Germany), placed at the top of each chamber. Oxygen evolution units were calculated after calibration according to the Winkler method [28]. Photosynthesis rate (P) was calculated from the difference between final and initial O_2_ measurements (∆O_2_) for each session. Photosynthetic efficiency (i.e., slope at the light limited region, α), irradiance compensation point (E_C_), saturation irradiance (E_K_), and maximal photosynthesis (P_max_) were calculated through a hyperbolic fit function [29]. Photosynthesis-Energy (PE) curve parameters were normalized to coral volume and water volume in each chamber. For volume measurements, the water volume of each chamber was measured using a cylinder and the coral volume on each tile was normalized by measuring the volume added to the cylinder after the tile had been introduced, and subtracting the tile volume from the total volume.

#### 2.5.2. Imaging-PAM 

Maximal quantum yield (F_v_/F_m_) was measured after 30 min of dark adaptation, and calculated using the maxi-version of Imaging-PAM instrument (Walz GmbH, Effeltrich, Germany). 

LED treatment and the control were tested under a 24-h cycle of constant light conditions (30 µmol photons m^−2^ s^−1^), followed by five hours of dark incubation. Light conditions and oxygen evolution measurements were similar to the PE curve setup. Samples of 100 mL of seawater were obtained from each chamber for alkalinity determination every 4–5 h and were stored at 4 °C for three days until analysis. We chose not to include the Yellow treatment in this experiment since the LED exhibited a greater effect than the Yellow, thus allowing us to increase technically the number of both LED and control replicates.

### 2.6. Calcification Rate

Calcification was estimated from changes in total alkalinity (A_T_) [30]. Alkalinity (µeq kg^−1^) was measured using a Compact titrosampler 862 (Metrohm, Herisau, Switzerland) and an automatic titrator (Mettler DL67). The seawater was slowly warmed to 25 °C, and each sample was filtered with a 0.2 μm filter into 30 mL duplicates. Calcification rates (μmoL CaCO_3 mL_^–1^ h^–1^) were then calculated using Equations (1) and (2):

ΔAlk = Alk_i_ − Alk_f_ (1)

(2) $$\;\;\;Calcification=\;\frac{\frac{\Delta Alk}{2}\times \left(V_{camber}-V_{coral}\right)}{T\;\left(hr\right)\;\times \;V_{coral}}\times 1000$$

where Alk_i_ (meq kg^–1^) is the initial alkalinity of seawater pre-incubation, Alk_f_ is the final alkalinity of seawater extracted from the chamber post-incubation, *V* is volume (mL), and *T* is the duration of incubation (h).

### 2.7. Statistical Analyses

All statistical analyses were performed using R [31]. Data were analyzed with Permutation ANOVA and t-tests using the R package {RVAideMemoire} [32]. In cases of small sample size and repeated measurements, we performed permutation tests in a linear mixed model, with repeats as a random effect, and followed by a pairwise comparisons package {predictmeans} [33].

## 3. Results

### 3.1. Settlement and Survivorship 

**Figure 1:**
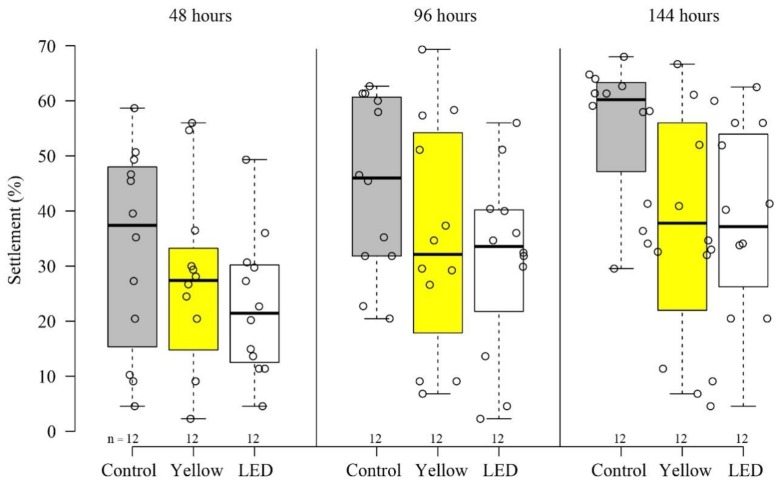
*S. pistillata* settlement (%) after 48, 96, and 144 h, under ambient conditions―control (gray), ‘Yellow’ (fluorescent lamp, yellow) light, and ‘LED’ (LED lamp, white) treatments. Each circle represents settlement (%) on an individual tile. Black center lines represent the medians; box limits represent the 25th to 75th percentiles of the data; whiskers extend to min and max values.

**Figure 2:**
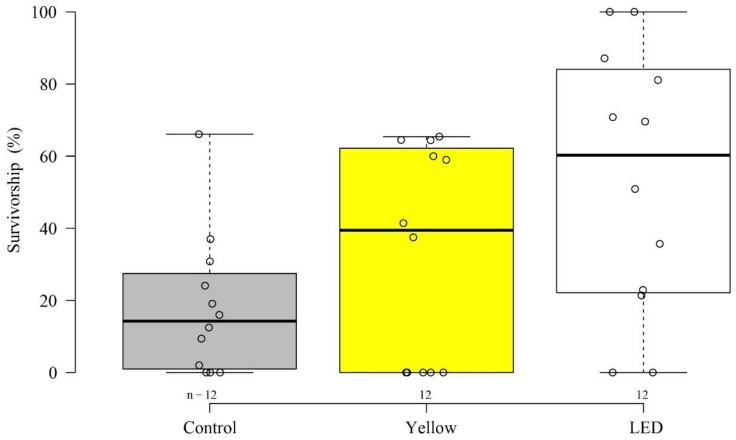
Survivorship (%) after one year (June 2017–June 2018) under ambient conditions—control (gray), ‘LED’ (LED lamp, white), and ‘Yellow’ (fluorescent lamp, yellow) light treatments. The circles represent survivorship on each settlement tile (*n* = number of tiles).

Our results revealed variability among the different illumination methods, in both settlement and survivability stages. After six days, the settlement percentages ± SE among the treatments (‘LED’―38% ± 4.9%, ‘Yellow’―37% ± 5.9%, and control―55% ± 3.8%) significantly differed (permutation ANOVA, *p* < 0.05) (Figure 1). The control showed a significantly higher settlement percentage than both ‘Yellow’ and ‘LED’ treatments (permutation t-tests, *p* < 0.05). There was no significant difference between the two illumination treatments, ‘Yellow’ and ‘LED’ (permutation t tests, *p* = 0.9). A differential effect of the light treatments was also found at the survivorship stage (permutation ANOVA, *p* < 0.05). The ‘LED’ treatment resulted in a higher but not significant survival percentage ± SE than both the control (permutation t tests, *p* = 0.054) and the ‘Yellow’― (permutation t tests, *p* = 0.15) (Figure 2) (‘LED’―53% ± 10.5%, ‘Yellow’―32% ± 8.6%, and control―18% ± 5.6%). The survivorship percentages of the planulae that had been introduced into the vessels under each treatment, developed into a coral colony, and survived for one year, were: ‘LED’―24%, ‘Yellow’―15%, and control―12%.

### 3.2. Growth Rate 

**Figure 3:**
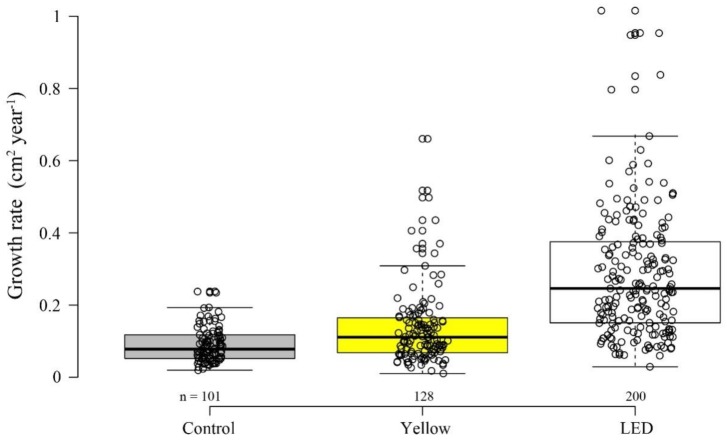
*S. pistillata* growth rate after one year (June 2017–June 2018) under ambient conditions— control (gray), and light treatments ‘Yellow’ (fluorescent lamp, yellow), and ‘LED’ (LED lamp, white). The circles represent individual colony growth rate (projected surface area; cm^2^ year^−1^) (n = number of colonies).

A similar trend to that found for survivorship was also found for growth rate. There was a significant difference in coral surface area among the treatments after one year (permutation ANOVA, *p* < 0.0001). Growth rate (cm^2^ year^−1^) under ‘LED’ was significantly higher than under both ‘Yellow’ and control (permutation t tests, *p* < 0.05) (Figure 3); while the ‘Yellow’ colonies showed a significantly faster growth rate than the control (permutation t tests, *p* < 0.05).

### 3.3. Photosynthesis and Calcification 

**Figure 4:**
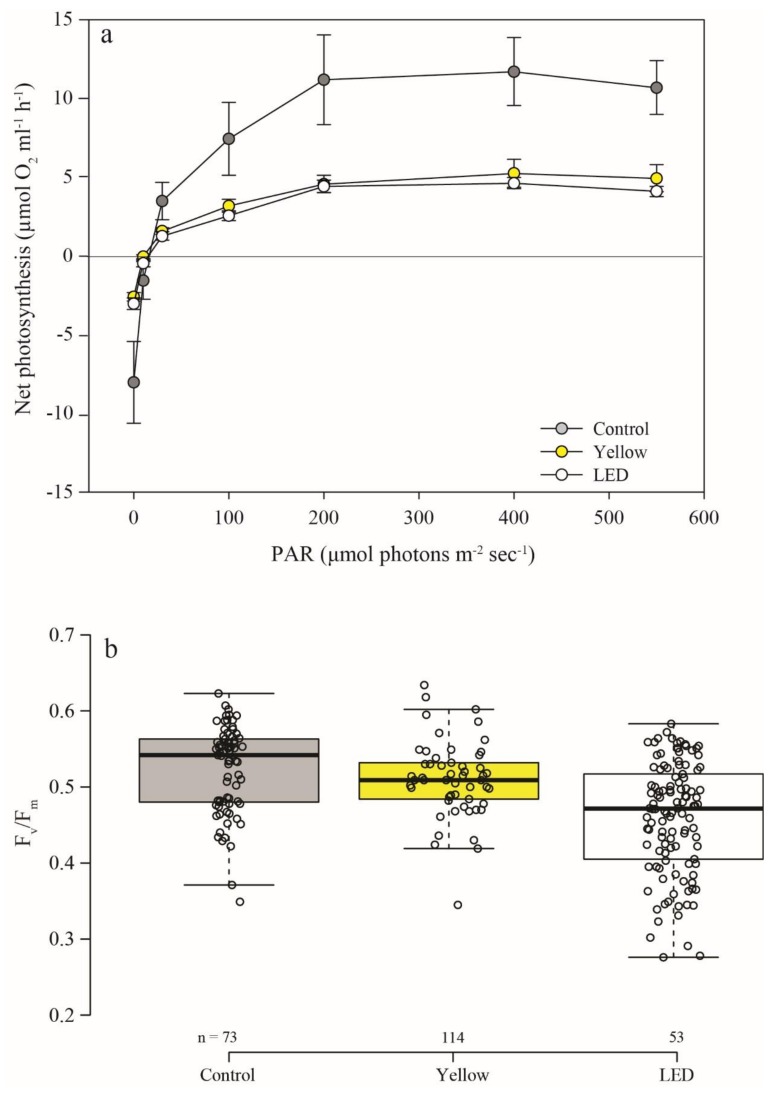
Photosynthesis efficiency of the experimental corals after one year. (**a**) PE (photosynthesis-energy) curve of the different light treatments—control (ambient, gray), ‘Yellow’ (fluorescent lamp, yellow), and ‘LED’ (LED lamp, white). Net-photosynthesis mean ±SE, under increasing light irradiance PAR (0, 10, 30, 100, 200, 400, 550 μmol photons m^−2^ s^−1^); *n* = 4 metabolic chamber measurements of individual tiles. (**b**) Photosynthetic maximal quantum yield (F_v_/F_m_) of the control (gray), ‘Yellow’ (yellow), and ‘LED’ (white). (*n* = the number of areas of interest measurement points).

The rate at which photosynthesis increased with light (slope at the light limited region—α) was calculated from the PE curves (Figure 4a) and found to be highest but not significant in the control corals (permutation ANOVA, *p* = 0.07) (Table 1). The control corals revealed a significantly higher potential maximum photosynthetic rate (P_max_) than both the ‘LED’ (permutation t-tests, *p* < 0.05) and ‘Yellow’ corals (permutation t tests, *p* < 0.05), with no significant difference between the two latter P_max_ (permutation t tests, *p* > 0.05). 

Photosynthetic maximal quantum yield (F_v_/F_m_) was also significantly affected by the treatments (permutation ANOVA, *p* < 0.01) (Figure 4b). The control corals showed the highest F_v_/F_m_. Furthermore, the ‘LED’ F_v_/F_m_ was significantly lower than in both control (permutation t-tests, *p* < 0.05), and ‘Yellow’ (permutation t-tests, *p* < 0.05). 

**Figure 5:**
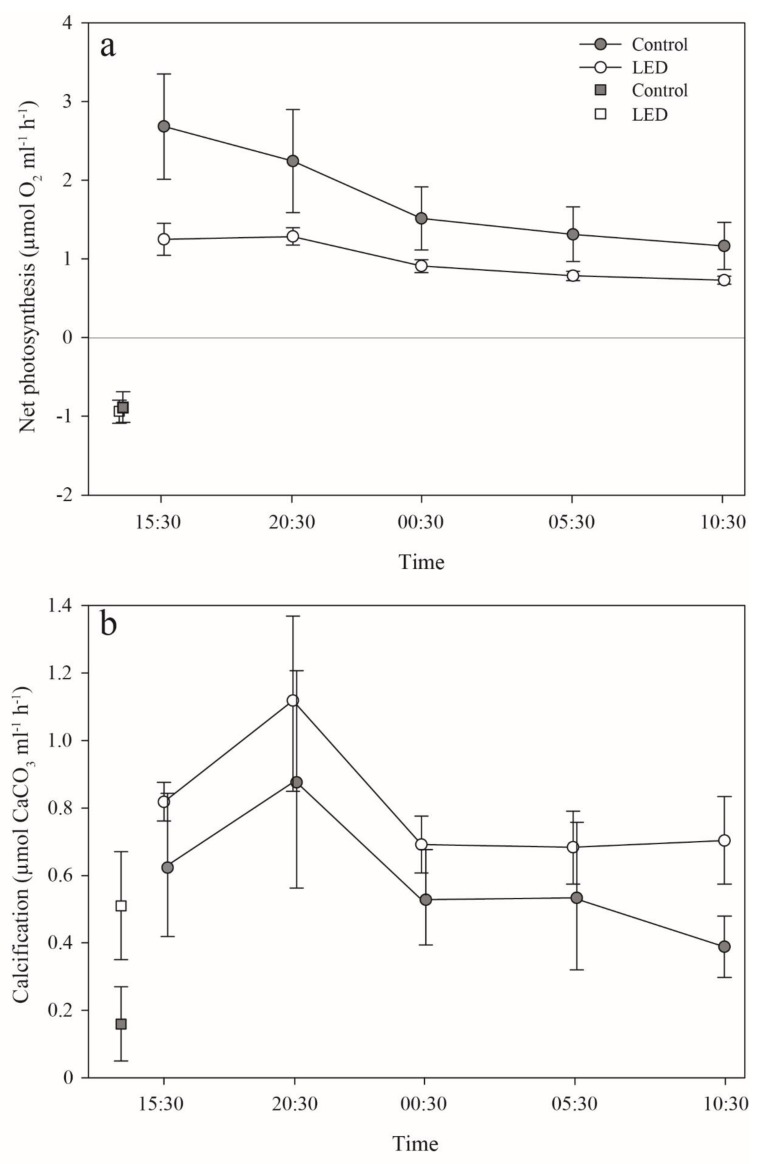
Net-photosynthesis and calcification rates during 24 h of incubation under dim light (30 μmoL photons m^−2^ s^−1^). (**a**) Net-photosynthesis rates (µmol O_2_ mL^−1^ h^−1^) mean ±SE. The squares represent the control (gray), and light treatment—‘LED’ (LED lamp, white) respiration rate under a 4 h dark cycle. (**b**) Calcification rates (µmol CaCO_3_ mL^−1^h^−1^) mean ±SE. The circles represent the control (gray) and light treatment—‘LED’ (white). The squares represent the control (gray) and light treatment—‘LED’ (white) calcification rates under a 4 h dark cycle. See further explanation for the statistics in the Results.

There was also a difference in photosynthesis among treatments in the long-term dim-light incubation experiment. Similar to the PE incubations, pairwise analyses revealed that the control corals demonstrated significantly higher net photosynthesis rates than the ‘LED’, throughout the entire 24-h incubation period (permutation t-test, *p* < 0.01) (Figure 5a). Photosynthetic rates measured during the first incubation (four hours) closely correlated to the rates measured during the short (24-h) incubation under 30 µmol photons m^−2^ s^−1^. However, the trend of photosynthesis throughout the 24-h incubation period decreased by 56% (the difference between the first and last sampling) in the controls, but by only 41% in the ‘LED’ treatment. Additionally, despite their higher net photosynthesis, pairwise analyses revealed that calcification of the control corals, although being lower than that of the ‘LED’ corals, did not differ significantly from the latter—at each sampling point (permutation t-test, *p* > 0.05) (Figure 5b). Calcification rates remained similar after 24 h in both treatments. Surprisingly, under four hours of dark incubation, the ‘LED’ corals, which were never exposed to the dark, calcified more than two-fold faster, but not significantly higher (permutation t-tests, P *p* > 0.05) than the control (0.51 ± 0.14 and 0.16 ± 0.11 µmol CaCO_3_ mL^−1^ h^−1^, respectively) (Figure 5b). However, the ratio between calcification in the light, in contrast to the calcification in the dark incubation, was higher in the control than in the ‘LED’ treatment (73% and 36%, respectively). Unlike the respiration difference measured for the short PE incubations, there was no difference in respiration between the two treatments after three hours of incubation (permutation t-tests, *p* > 0.05) (Figure 5a). We excluded the potential effect of the substrate tiles, since a control incubation with an empty tile taken from the control section of the experimental setup (ambient conditions), revealed no calcification (−0.0014 ± 0.0026 µmol CaCO_3_ ml^−1^ h^−1^) throughout the incubation period. 

In order to perform as many repetitions as possible and to strengthen the statistics, and in light of the fact that the two lighting methods treatments (LED and Yellow—fluorescence) exhibited similar photosynthesis efficiency (Figure 4), we chose to perform the metabolic chambers tests only for the LED and the control groups. Hence, the fluorescence (Yellow) does not appear in Figure 5.

## 4. Discussion

Finding a suitable settlement site is a crucial process for the recruitment and survivorship of marine sessile invertebrate larvae [4], and thus may directly determine the distribution of different species at specific locations [14]. The findings from the present work have enabled us to elucidate the physiological and ecological effects of two different types of common urban artificial lighting on crucial life stages and physiology (i.e., settlement, survivorship, growth rates, calcification rates, and photosynthetic efficiency) of the coral *Stylophora pistillata*. Over the past few decades, light source diversity has increased [34]. This trend, together with the adoption of lighting technologies presenting a broader spectrum, i.e., featuring ‘white’ light (e.g., LED lighting methods) specifically, is becoming more common [25]. As a result of the LED short wavelengths combined with the water’s physical characteristics [13], an ever-increasing area of the reef and of its specific coral community that thrives at shallow depths (0–30 m), is expected to experience greater disturbance from such artificial illumination at night.

The results of our settlement experiments indicate that light pollution is likely to reduce the percentage of *S. pistillata* planulae settling on the substrate (Figure 1). *S. pistillata* is currently the most common recruited species among the stony corals in the shallow depths in the GoE/A [27]. Since the shallows are in close proximity to the shore, these corals are more exposed to such artificial illumination [13], in addition to other anthropogenic disturbances. This, in the long term, may be potentially harmful to certain coral species. 

Bolton et al. [35] demonstrated a dramatically direct effect of artificial lighting on the predatory behavior of fish, which increased their pace of predation at night; as well as the indirect effects of this lighting on sessile assemblage structures (e.g., barnacles, ascidians, and polychaetes). This change in substrate may have an indirect but crucial effect on coral recruitment and resilience (e.g., new settlers). Such changes in benthic community composition can play an important role in coral recruitment dynamics [36]. Hence, in the long term, along with the increasing use of artificial light, and specifically LED lights, *S. pistillata* settlement patterns may be interrupted.

The “recruitment-limited” theory for open-water marine populations was first postulated for coral-reef fishes [37], and later accepted as applying to many marine sessile organisms possessing a dispersive larval stage [38,39]. This theory predicts that population dynamics will be primarily driven by the magnitude and variation in the supply of larvae to the population, rather than by processes acting post settlement; as well as driven by ecological phenomena such as fish predation during coral spawning. This may be an important source of coral larvae mortality [40], which has the potential to increase under light pollution. Hence, changes in the coral larvae natural light regime may result in the degradation of a coral community as a consequence of the reduced supply of larvae. Given that coral larvae frequently travel between reefs [41], a large spatial variation is expected in coral larvae availability due to the light pollution regime, on the scale of both entire reefs and within-reef habitats. Moreover, globally, asynchronous planulae release by different coral species [9,42], due to slight changes in the light regime, may lead to reproductive isolation and prevent gene flow between shallow coral reefs communities that are exposed to light pollution globally [42,43,44]. Although it has not yet been proven, such asynchronization may be affected by light pollution, leading to changes in the proportion of recruited brooding versus broadcast-spawning species [45]. 

Our findings for the survivorship stage revealed an opposite trend, in which the artificially-illuminated corals demonstrated higher percentages of survivorship (Figure 2). Similarly, under illuminated conditions, they demonstrated significantly higher growth rates than their counterparts in the control (Figure 3). These survivorship and growth rate results indicate the potential advantage of possessing an additional photosynthetic energy flux, which in this case is acquired at night [46]. Previous studies have engaged with the question of increasing plant production capacity through the use of controlled artificial lighting systems [46,47]. Their findings revealed how LED can mimic natural light to conduce to the growth and development of photosynthetic organisms. Nonetheless, the survivorship percentages between the light treatments and the control in the present study were not significant.

Despite the higher survivorship, there was a lower photosynthetic efficiency pattern in both light-polluted coral treatments than in the control. The lower photosynthetic efficiency (i.e., lower slope—α) of the symbionts in the corals exposed to artificial lighting (Figure 4, Table 1) could be due to the photosynthetic apparatus being either not fully activated normally, or inactivated. These differences in photosynthetic efficiency (α) between the two treatments and the control could be due to the higher respiration rate of the latter. In addition, the lower slope (α) resulting from the light treatment may indicate possible damage to RuBisCO efficiency and CO_2_ assimilation ability [48], despite the increase in light intensity; or could be due to the low amount of RuBisCO present [49]. RuBisCO is known to be a limiting factor mainly at high light intensity, i.e., mostly limiting the maximum potential photosynthetic rate (P_max_).

There was a difference between the control and light-polluted treatments in PE curves and long dark-incubation respiration (Figure 4a and Figure 5a). The PE curves, measured during daytime, lasted 20 min at each light intensity (or dark); while the long dark-incubation period was during the night, reflecting the corals’ normal daily cycle. It is possible that the faster respiration of the control corals was measured in the pre-PE dark period, because their photosynthesis during the day was higher compared with the ‘LED’ corals. This difference between night and day respiration in the control corals was not evident in the ‘LED’ corals, possibly implying a disruption of their natural cycle. Recently, Ayalon et al. [50] reported a significantly lower photosynthesis performance (decreasing levels of PSII electron transport rate—ETR) of *Acropora eurystoma* and *Pocillopora damicornis* when exposed to LED lights. Moreover, they demonstrated that the blue and the white LED spectra demonstrated a more negative impact on coral physiology compared to the Yellow LED. Thus, similar to previous studies, the differences found between our control and light treatment photosynthesis performances may reflect the variability that exists between the effects of the different artificial lighting methods on a coral’s basic physiology parameters.

Interestingly, despite the lower photosynthetic efficiency and rates of the ‘LED’ corals throughout the entire 24-h incubation period, calcification was higher than in the control corals (Figure 5). This might be explained by the light-enhanced calcification (LEC) process, which leads to variation in growth rates. Even though the interrelationship of hermatypic coral-calcification and photosynthesis has been determined as a general concept [51], previous studies have shown the potential of LEC to be directly affected by light without the mediation of photosynthesis [52,53]. Furthermore, Cohen et al. [52] suggested that a blue light signal and its receptors in animals may be involved in the enhancement of calcification by hermatypic corals. The faster growth of the corals under the LED treatment supports the assumption that this trend may be due to the presence of LED at night. Thus, the observed increase in growth rates of corals illuminated by LED light may be explained by the absorption of blue light by several blue light photoreceptors found in the coral host [54]. Such photoreceptors have been discovered in several *Acropora* spp. corals and identified as cryptochromes and opsins, which absorb mainly blue light [12]. However, we do not know how a longer light exposure of the corals to LED might affect their physiology. This question, in addition to estimating the time it takes for light to stimulate calcification at night, should be addressed in future studies. 

Rocha et al. [55] examined the effect of the artificial light spectrum on growth performance of cultured scleractinian corals following exposure to identical photosynthetically-active radiation (PAR) intensities. They tested this by calculating the effect of different light spectra delivering identical PAR by means of fluorescent lamps (used as a control treatment), Light Emitting Plasma (LEP), and Light Emitting Diode (LED) on the photobiology of two scleractinian corals: *Acropora formosa* and *S. pistillata*. The particular light spectrum significantly affected coral growth of both species. *A. formosa* cultured under LED presented a specific growth rate 99% higher than conspecifics grown under fluorescent illumination (control). Wijgerde et al. [56] exposed *Galaxea fascicularis* to similar LEP and LED light intensities. Interestingly, under relatively low irradiance (40–60 μmol m^−2^ s^−1^), the growth rate of these corals was higher under the LED treatment. This trend was reversed when light intensity was increased. Such a trend may be a result of the LED higher blue peak. These findings may explain the higher growth rate under the light treatments, even though the artificial night-time light levels were very low (i.e., below the compensation point; Figure 5a). In addition to the effect of blue light on coral physiology, it was found that red light also has a considerable potential negative effect on *S. pistillata* health and survivorship, even under the combination of blue and red wavelength peaks (452 and 665 nm; [57]). The two light treatments in the current experiment also yielded a portion of red light. An additional potential negative trend has recently been found by using transcriptome analysis under LED treatment to compare corals growing under natural light cycles and under light pollution conditions [20]. Disorders expressed as alterations in gene expression pathways (cell proliferation, organismal injury and abnormalities, and reproductive system disease genes) were demonstrated to be a direct result of the exposure to light pollution [20]. That study revealed many altered pathways that had resulted in cell-cycle progression, cell proliferation, survival, and growth in the long term.

The various city light sources i.e., roadside lights, car headlights, street lighting, and other city lights, especially those located next to roads and other marine structures such as ports and oil jetties [13], may interrupt natural processes (e.g., photosynthesis and calcification). Bennie et al. [58] contended that in practice, the measurable effects of light on carbon fixation in terrestrial vegetation are likely to be limited to situations in which the leaves are in very close proximity to a light source, or when artificial lighting is introduced into naturally dark situations such as cave systems. A similar situation can be found at those reef sites where a naturally dark regime becomes illuminated by permanent artificial lights. However, no evidence of a photosynthesis process taking place under low artificial lighting was found in our study. Thus, although the artificial lighting effect is seemingly advantageous, i.e., higher survivorship and rapid growth rate, it will not necessarily contribute to the thriving of a particular species, especially if such lighting is detrimental to that species’ ability to recruit, mainly due to a reduced supply of larvae and lower settlement success. The question, however, is how does the added survivorship relate to the reduced settlement—do they balance each other out? Based on the “recruitment-limited” theory [37] and the fact that during the settlement stage the planulae had remained in closed vessels, thus preventing any possibility of settling elsewhere, we conclude that in a natural reef under a light pollution regime, the ‘recruitment equilibrium’ will become unbalanced. We should therefore consider the overall consequences of light pollution on coral-reef physiology and ecology, and avoid drawing unequivocal conclusions regarding each of the coral life phases separately.

Understanding the impacts of light pollution at night on coral species requires knowledge of the intensity, spatial pattern, spectral distribution, duration, and timing of the artificial lighting to which corals are exposed. Hence, specific crucial aspects pertaining to coral life-traits (e.g., recruitment, settlement, and survivorship) should also be examined in a variety of coral species. In addition, it is necessary to determine the importance of recruitment relative to post-settlement survivorship related to processes such as predation [35], competition, and disturbance [59] under a light pollution regime. Moreover, in addition to methods such as determining gene expression in relation to advanced life stages, the effect of artificial lights on zooxanthellae acquired at early life stages of larvae or juvenile coral colonies, as well as skeleton structure, ecological studies (e.g., reproductive fecundity) are also needed in order to address the effects of light pollution on coral physiology and ecology.

## 5. Conclusions

This work presents for the first time the early life-history traits (e.g., settlement, growth rate, and survivorship) and photosynthetic efficiency patterns of a coral species under a long-term light pollution regime. With the increase in the human population and technological developments, we are witnessing a change in the night-time lighting regime as a result of different lighting methods (i.e., substitution of the high-pressure sodium lamps (HPS) with LEDs). ’Modern’ lighting, as shown in this study, not only penetrates deeper into the water body [13], but also significantly adversely affects the physiology and the critically fragile early stages of coral settlement. We therefore strongly recommend an appropriate use of lighting methods that will minimize the disturbance to marine coastal environments. This can be achieved by practical solutions such as using light sources of minimal adverse environmental effects (e.g., HPS and/or fluorescence lights instead of LEDs, characterized by short-energetic wavelengths); or alternatively, using LED lamps with lower Kelvin ratings (i.e., lower intensity and spectrum; specifically reducing the blue peak). 

## Figures and Tables

**Table 1:** PE (photosynthesis-energy) curve parameters. ‘Dark’ (control), ‘Yellow’ (fluorescent), and ‘LED’ (LED) represent the PE curve parameters of the different treatments. Parameters after fitting to a nonlinear hyperbolic tangent function: R^2^ = coefficient of determination; slope (α) = maximum light utilization coefficient; E_c_= Compensation point; E_k_ = minimum saturating irradiance; P_max_= maximum potential photosynthetic rate; SE = standard error of the mean.

Treatment	R^2^	Slope (α)	SE	E_c_	SE	P_max_	SE	E_k_	SE
**Control**	0.94	0.49	0.19	22.57	6.53	18.77	4.59	58.80	23.89
**Yellow**	0.96	0.22	0.07	13.00	0.61	8.10	0.80	38.46	7.77
**LED**	0.93	0.19	0.04	19.39	2.07	7.01	0.62	44.50	6.75

## Supplementary Materials

The following are available online at https://www.mdpi.com/2076-2607/8/2/193/s1, Figure S1: Multiyear temperature oscillations at the water tables setup. Table S1: Statistical values obtained for the settlement, survivorship, growth rate, calcification rate, net photosynthesis rate and PE (photosynthesis-energy) curve parameters.
