# Supplementary material for: Effects of Light Pollution on the Early Life Stages of the Most Abundant Northern Red Sea Coral
Source: Microorganisms. 2020 Jan 31;8(2):193. doi: 10.3390/microorganisms8020193 (PMC7074826; doi:10.3390/microorganisms8020193)
Supplement: Supplementary file 1 [file microorganisms-08-00193-s001.pdf]

SUPPLEMENTARY MATERIAL

Effect of light pollution on the early life stages of the most abundant northern Red Sea coral

Raz Tamir[1,2], Gal Eyal[3,4], Itay Cohen[2,5], and Yossi Loya[1]

1. School of Zoology, George S. Wise Faculty of Life Sciences, Tel Aviv University, ISRAEL

2. The Interuniversity Institute for Marine Sciences in Eilat, ISRAEL

3. ARC Centre of Excellence for Coral Reef Studies, School of Biological Sciences, University of Queensland, AUSTRALIA

4. The Mina & Everard Goodman Faculty of Life Sciences, Bar Ilan University, ISRAEL

5. Department of Oceanography, The Institute of Earth Sciences, The Hebrew University of Jerusalem, ISRAEL,

Corresponding author: R. Tamir, The Interuniversity Institute for Marine Sciences in Eilat, ISRAEL

Email: raztamir6@gmail.com.

TABLE 1S. Statistical values obtained for the settlement, survivorship, growth rate, calcification rate, net photosynthesis rate and PE (photosynthesis-energy) curve parameters. 'Dark' (control), 'Yellow' (fluorescent), and 'LED' (LED) represent the the different treatments. PE Parameters: F_v/F_m, slope (α) = maximum light utilization coefficient; P_{max}= maximum potential photosynthetic rate.

	**Permutation ANOVA**		**Permutational t-tests**		
	F (df)	***P***	***Dark - LED***	***Dark - Yellow***	***LED - Yellow***
**Settlement**	3.5 (2,33)	0.043	0.042	0.042	0.9
**Survivorship**	3.42 (2,33)	0.047	0.054	0.46	0.15
**Growth rate (cm^2/year)**	73.32 (2,427)	0.001	0.003	0.006	0.003
**F_v/F_m**	24.23 (2,233)	0.001	0.003	0.218	0.003
**Slope (α)**	2.53 (2,11)	0.07	0.19	0.71	0.71
**Pmax**	7.626 (2,11)	0.027	0.018	0.2	0.42
**Calcification rate**	NA		0.1	NA	NA
**Net photosynthesis rate**	NA		0.8	NA	NA

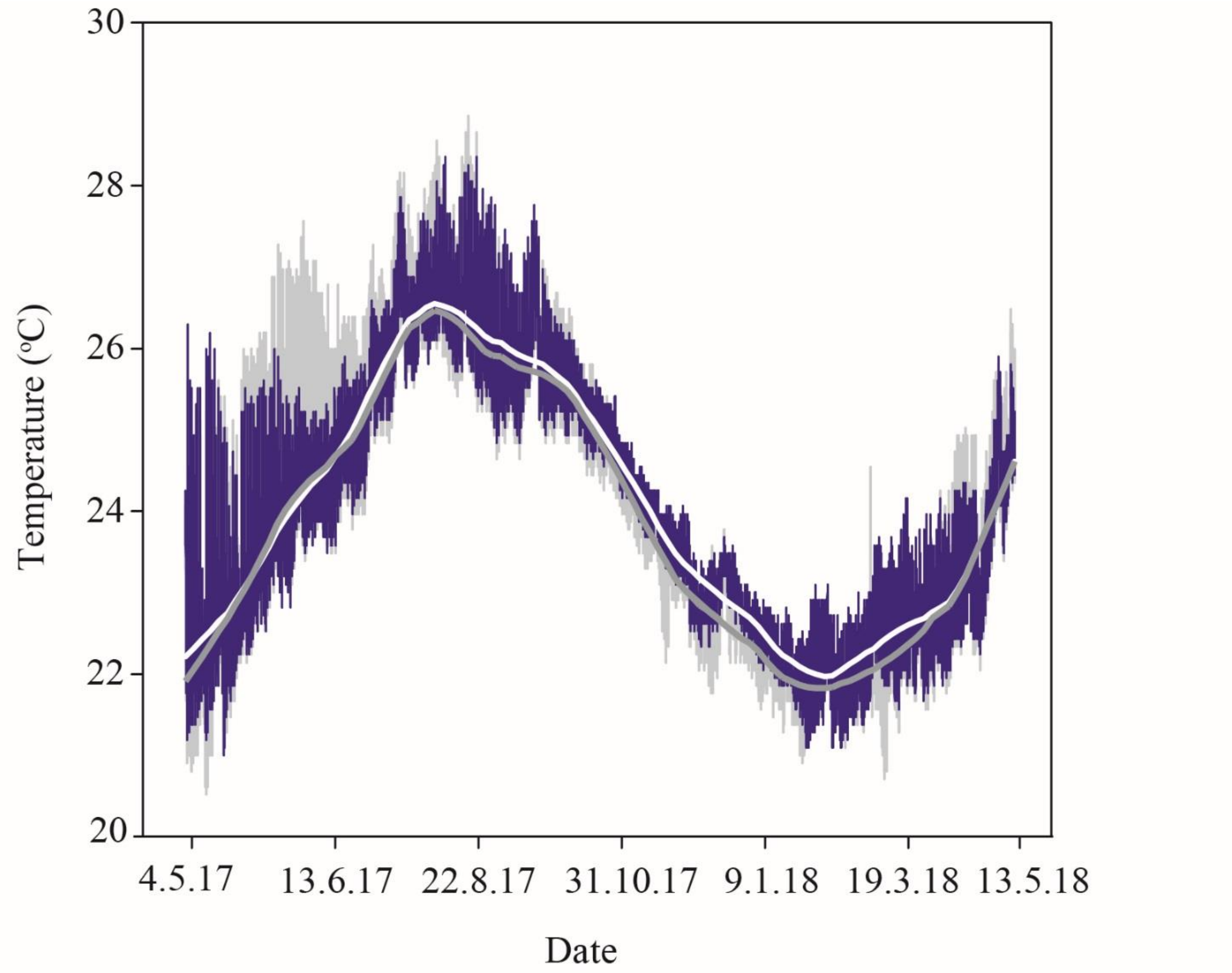

FIGURE 1S. Multiyear temperature oscillations at the water tables setup. Water temperatures are represented by blue line (LED) and gray line (control) in 10-min interval measurements. White line shows the running median of the LED measurements and the dark-gray line indicates the control.

Does light pollution affect the deep reef?

Of the various ecological niches in which stony corals are able to establish themselves, the Mesophotic Coral Ecosystems (MCEs) are where ambient light is most limited [1] as a result of their great depth (>30 m). In view of the global shallow-water coral-reef ecosystem degradation, MCEs are gaining increasing interest [2]. Hence, in terms of these issues, due to the relatively low light intensity at those depths, we assume negligible effect on coral physiological and photosynthetic coral-reef scientists and managers [2-3]. This is because they are linked both physically and biologically to their shallow-water counterparts, and thus have the potential both to be refugia for shallow coral-reef taxa and to be a potential source of larvae that could contribute to the resiliency of shallow-water reefs (i.e., the Deep Reef Refugia Hypothesis (DRRH); [4]. Hence, in terms of these issues, due to the relatively low light intensity at those depths, we assume negligible effect on coral physiological and photosynthetic processes. Nevertheless, the ability of artificial light to penetrate into the mesophotic water column [5] and the ability of the deep reef to serve as a refugium for shallow species and vice versa, should be farther examined. Indeed, studying the effect of the light pollution phenomenon on the connectivity between shallow and mesophotic reefs [6], may lead to findings that will need to be taken into account in the future.

REFERENCES

1. Lesser, M. P., Slattery, M., & Leichter, J. J. (2009). Ecology of mesophotic coral reefs. *Journal of Experimental Marine Biology and Ecology*, 1, 1-8.
2. Loya, Y., K. A. Puglise, T. C. L. Bridge (2019). Mesophotic coral ecosystems. Springer, New York.
3. Loya, Y., G. Eyal, et al. (2016). Theme section on mesophotic coral ecosystems: advances in knowledge and future perspectives, Springer.

4. Bongaerts, P., Ridgway, T., Sampayo, E. M., & Hoegh-Guldberg, O. (2010). Assessing the 'deep reef refugia'hypothesis: focus on Caribbean reefs. *Coral reefs*, 29(2), 309-327.
5. Tamir, R., Lerner A., et al. (2017). The spectral and spatial distribution of light pollution in the waters of the northern Gulf of Aqaba (Eilat). *Scientific Reports*, 7, 42329.
6. Holstein, D. M., Paris, C. B., Vaz, A. C., & Smith, T. B. (2016). Modeling vertical coral connectivity and mesophotic refugia. *Coral Reefs*, 35, 23-37.
